# The Institutional Worker

**Published:** 1920-03-27

**Authors:** 


THE
### INSTITUTIONAL WORKER
**Being a Special Supplement to "The Hospital"**

### OUR BUREAU OF INFORMATION.

**Rules for Correspondents.**

1. Every letter must be accompanied by the coupon to be cut from the back cover (inside page) of The Hospital, current issue, and must contain the name and address of the correspondent with pseudonym for publication if desired. Replies by post cannot be given save under exceptional circumstances at the Editor's discretion.
2. Letters from Approved Homes in reply to special needs published in the Bureau should state terms and full particulars, and be sent prepaid under cover to the Editor of the Bureau with name written across coupon for identification.
3. Proprietors of Homes which have not yet been entered on the List of Approved Homes, but have spare accommodation likely to suit special needs, are invited to write for an application form for registration. The fee for registration, which includes two announcements of the Home in the Bureau and other privileges, is 10s.
4. All communications to be addressed to the Editor of The Hospital, 28 Southampton Street, Strand. London, W.C. 2, and marked "Bureau of Information."

### INSTITUTION FACTS AND FIGURES.

### The Question Box.

### A NURSE'S VENTURE AS OPTICIAN.

The question was:

**A trained nurse with long experience in an eye hospital, who has taken the certificate of the British Optical Association, is desirous of setting up business in a flourishing provincial town. Give suggestions and advice as to size of shop, its fittings, capital required, staff required, and the best method of making the shop known by advertising or otherwise.**

The source for the information required in the answer to this question is obtained mainly from an article which appeared some time back in "Women's Employment," the organ of the Central Bureau for educated women workers, from the pen of Mr. J. H. Sutcliffe, Secretary and Director of Examinations, British Optical Association.

In setting up practice the woman optician requires just about the same amount of capital, nerve, and business capability that would be required by a young dentist. It would be foolish to open a large shop or to advertise widely, in imitation of some of the bigger opticians. I should recommend a couple of ground-floor offices in a central position in some provincial town. Very little stock would be required—in fact, modern good work requires the fitting and manufacture of special spectacles for each client. If there was sufficient capital to pay rent and the cost of living for the first twelve months, success should follow. Expenses would, of course, be much less if the woman optician could, at any rate to begin with, use two rooms in her own home for consulting and waiting rooms. The drawback to this arrangement is that very often the business is not taken seriously enough for it to become profitable.

In whatever way the business is started, the initial outlay required is less than in any other semi-professional trade. The chief qualification required (other than professional competence) will be business acumen, such as is possessed by the average Frenchwoman who controls her husband's cash desk. Assuming always the possession of the necessary technical ability, the same amount of business brains that would enable a woman to run a successful tea-room ought to allow her to make a good living as an optician. Without that ability failure is certain.

The clients one meets as a rule are decent, cash-paying people. In the case of a woman optician settling down in a provincial town, they would come from the middle classes, and would probably consist of women and children sent by the local practitioners. I cannot quote from precedent, as the number of women who have taken up this work are few, but I think that a woman optician with the qualifications I have outlined, ought soon to be in possession of a clear income of not less than £300 a year. By engaging competent assistants more than that is both possible and probable. The work is light and fascinating, and, it may be added, that it is one of the few occupations where age is an advantage rather than a drawback.

An Army Spectacle Scheme was inaugurated in January 1916, spectacle centres being established at various depôts and hospitals, both at home and abroad, and at each of these was posted a specially enlisted optician. Now that these men have been—or are being—demobilised, it is quite possible that there may be a demand for the services of capable women in their places at the various hospitals. S. C. H.

### THE CARE OF THE FACTORY WORKER IN THE U.S.A.

The U.S. Department of Labour (Bureau of Labour Statistics) publishes a useful booklet, "Welfare Work for Employees in Industrial Establishments in the United States."

Far as we have advanced in this country as regards schemes of industrial betterment, we find that the best American firms are still far ahead of any, except, say, three or four factories in Great Britain. America goes more thoroughly into the matter, and there are no half-measures of welfare undertaken. Such things as construction, equipment, upkeep, and extent of the welfare provisions are on a much more lavish and scientific scale than in the Old Country, and the American employer is firmly convinced that welfare is a paying proposition.

We note particularly and with special interest the superior nature of the drinking-water and bathing facilities, the provision of *paper* towels and liquid soap, and the sanitary custom of having wire lockers installed in many of the cloak-rooms. Rest periods and sick leave with pay are granted in the case of some firms, and others make special provision for the treatment of tubercular employees.

We quote in full the list of equipment considered adequate for a factory emergency hospital, as it may be found useful by those interested in the health side of industry :—

List of Equipment for Emergency Hospital.

- 1 Water and instrument sterilizer.
- 1 Metal white-enamelled operating-table, complete with cushions.
- 1 Metal white-enamelled physician's chair, with headrest for treatment of the eyes.
- 1 Metal white-enamelled dressing-case, top 36 inches long.
- 1 Metal white-enamelled three-section dust-proof supply cabinet, each section 12 inches by 12 inches by 5 feet, with shelves.
- 1 Metal white-enamelled electric-light stand.
- 1 Electric fan.
- 1 Metal white-enamelled medical and surgical cabinet.

(*Continued on p. 2, col. 3.*)

### Question for March.

### WIDENING THE AREA OF HOSPITAL SUPPORTERS.

**Elaborate a plan for obtaining the financial assistance of members of the middle classes who do not at present contribute to hospital funds and who cannot afford to become annual subscribers. Outline methods of working, without involving an increase of staff.**

**(A minimum payment of 10s. 6d. will be made for each published answer.)**

RULES.

The following rules must be observed:—

1. Contributions must be written on one side of the paper. Conciseness and terseness are desirable features. The MS. must bear the name and address of the sender and be accompanied by coupon to be cut from the back cover (inside page) of the current issue of The Hospital. A pseudonym must be chosen if the name is not to be published.

2. Contributions must be addressed to the Editor of The Hospital Institutional Worker Supplement, 28 & 29 Southampton Street, Strand, London, W.C. 2, should reach him before the end of the current month, and be marked in the left-hand corner "Facts and Figures."

### QUESTIONS INVITED.

In connection with our Question Box, we offer every month five shillings each for the best questions which are sent in for consideration. The questions may be on any institutional subject and concern any department, from the secretary's to the porter's, or the matron's to the domestic staff; the one essential is that they must be practical and deal with points that have a definite relation to hospital or institutional administration, upkeep, finance, or management. Points relating to artificers' work, housekeeping, the laundry, out-patients, and other practical matters will be welcome. It is hoped that thus, when difficult or doubtful points arise in the course of their work, institutional workers will be encouraged to put them in the form of a question and send them to the Editor, The Hospital Bureau of Information, 28 & 29 Southampton Street, Strand, London, W.C. 2, marked "Question Box."

The best questions will be published in due course, and our readers will be given the opportunity of answering them. Thus all institutional workers may be able to co-operate with the view of helping the work and smoothing the difficulties of each other. In short, we want the Question Box of the *Institutional Worker* to be freely used by every worker habitually.

### ENQUIRIES AND ANSWERS.

### *EMPLOYMENT AND TRAINING.*

#### Filling in the Waiting Period.

A post as wardmaid would in no way help towards your general training, and we know of no hospital employing part-time probationers. We suggest that you enter either a children's hospital or a special hospital, such as the Royal Chest Hospital, City Road, E.C. 1, for two years' training. Apply to the Matrons of the following children's hospitals: Alexandra Hospital for Children with Hip Disease, Queen Square, Bloomsbury, W.C.; The Infants' Hospital, Vincent Square, Westminster, S.W.; and Lord Mayor Treloar Hospital for Crippled Children, Alton, Hants. The latter gives a three years' training. If you prefer dispensing write for particulars of training, etc., to Miss Buchanan, Pharmaceutical Chemist, Gordon Hall, Gordon Square, W.C.—N. M. C.

#### Massage Training for Services.

If you are a fully trained nurse and over twenty-four years of age you could obtain free training in massage, Swedish remedial exercises, and medical electricity in exchange for services at the National Hospital for the Paralysed and Epileptic, Queen Square, London, W.C. At the same time you would receive a salary of £2 a month, board, lodging, and uniform. You would be required to give two years' service.—J. B.

#### Special Terms for Trained Nurses for Midwifery Training.

The following institutions offer special terms to trained nurses for midwifery training: Queen Charlotte's Lying-in Hospital, Marylebone, N.W. 1; East-End Mothers' Lying-in Home, 396 Commercial Road, Stepney, E.; and Fulham Midwifery Training School, St. Mary's Nursing Home, Parson's Green. We suggest that you do not obtain any books upon this subject until you have chosen your training-school, and then ask your principal what you will require.—Stellate.

#### A Nursing Service.

The Pensioners' Nursing Service is a service composed of nurses who attend disabled soldiers in hospitals specially set aside for that purpose. Apply to the Matron-in-Chief, Miss Davies, R.R.C., 14 Great Smith Street, Westminster, S.W. 1.—Terris.

#### C.M.B. Examinations.

The new regulations in regard to the dates at which examinations under the Central Midwives' Board will be held—*i.e.*, every two months in London, and every two months alternately in Birmingham and Bristol, Manchester, and Liverpool, and Leeds and Newcastle—do not come into force until 1921. The address of the Central Midwives' Board is 1 Queen Anne's Gate, Westminster, S.W.—Enquirer.

### *MISCELLANEOUS.*

#### Arm Sling.

So many devices by nurses and others for the comfort of patients come to our notice that we judge that if every one were patented or placed on the market financial loss would in most instances result. We would not, therefore, advise you to take out a patent for your special sling, even if you found it feasible. You might perhaps submit it to a surgical-appliance maker, and if he should consider the device a valuable one you could possibly make some arrangement with him for placing it on the market.—A. C. W.

### THE CARE OF THE FACTORY WORKER IN THE U.S.A.

(*Continued from p.* 1.)

1 Metal white-enamelled two-basin stand, with instrument tray (revolving).
1 Small roll-top desk.
1 Hospital bed, including mattress and pillows.
1 Three-panelled folding screen for bed.
1 Revolving stool (metal, white enamelled.)
2 Metal white-enamelled chairs.
1 Standard porcelain enamelled foot-bath.
1 Justrite pail.
1 Electric heating blanket.
2 Electric heating pads.
1 Bandage scissors, 6 inch.
1 Mouth gag.
1 Razor.
1 Military hypodermic syringe.
6 Scalpels.
2 Bistouries (straight).
1 Bistoury (curved).
2 Dull-pointed scissors, 5 inch (straight).
2 Dull-pointed scissors (curved).
1 Suture scissors.
1 Grove director.
4 Probes.
1 Curette.
1 Lister's bone forceps, 7½ inches.
24 Curved needles.
1 Needle holder.
2 Plain tissue forceps.
2 Mouse-tooth forceps.
1 Double-pointed eye spud.
1 Eye magnifying glass.
1 Foot rest.
1 Kelley pad.
12 Tubes, emergency, catgut.
6 Reagent bottles, 4 ounce, glass stoppers.
2 Two-quart porcelain-enamelled pitchers.
2 Basins.
1 Pus basin.
6 Glass syringes.
12 Medicine droppers.
1 Esmarc bandage.
24 Towels.
2 Army and Navy tourniquets.

